# Incisionless Pubovaginal Fascial Sling Using Transvaginal Bone Anchors for the Treatment of Stress Urinary Incontinence

**DOI:** 10.1100/tsw.2004.90

**Published:** 2004-06-07

**Authors:** Sandip P. Vasavada, Craig V. Comiter, Shlomo Raz

**Affiliations:** The University of California, Los Angeles School of Medicine, Los Angeles, CA, USA

## Abstract

Introduction: Bladder neck suspension (BNS) for stress urinary incontinence (SUI) can have significant morbidity, including bleeding, infection and pain. In an effort to reduce this potential morbidity, we have devised a technique which provides the same suburethral support as a standard anterior vaginal wall sling (AVWS), but without a vaginal or suprapubic incision. We describe this minimally invasive technique. Methods: From April 1998 to February 1999, 85 women underwent an incisionless suburethral fascial sling procedure. A transvaginal bone drill was used to place a bone anchor loaded with #1 prolene suture into the inferior aspect of the pubic bone on either side of the urethra. A subepithelial tunnel was created at the level of the bladder neck. A 2 x 7 cm segment of cadaveric fascia lata was placed through the subepithelial tunnel. The sutures were passed through the fascia 5mm from either edge, effectively creating a 6.0 cm sling. Finally, the sutures are tied up to the pubic symphysis.Results: Follow-up was via a self-administered questionnaire and patient interview. Recurrent SUI was noted in 2/85 (3%). New onset urge incontinence was present in 4/85 (5%). Permanent urinary retention has not occurred in either group. All procedures were performed on an outpatient basis and no operative complications occurred.Conclusions: Early results for the incisionless sling compare favorably with the long term results for the AVWS. This minimally invasive approach has thus far not been associated with any significant complications. Elimination of the vaginal and suprapubic incisions has not compromised efficacy, and appears to reduce the incidence of urge incontinence. Long term follow-up will establish the lasting efficacy of this novel surgical technique.

## INTRODUCTION

Vaginal sling surgery has been part of the urologist’s armamentarium since the mid 1900ís when it was first described by Von Giordano and Aldridge (1). The early descriptions of the pubovaginal sling surgery entailed placement of autologous tissue underneath the bladder neck and suspending it superiorly. The early procedures were often complicated by erosion, infection, bleeding and fistula formation. Nonetheless, its success in managing more complex causes of incontinence allowed it to gain a place in the repertoire of many incontinence surgeons. Still, many believed it should be reserved for more complicated cases of incontinence and accordingly, used it sparingly. As more recent data has shown, transvaginal needle suspensions have worked well with stress urinary incontinence when associated with urethral hypermobility alone (2). The limitation of these approaches has been in patients with intrinsic sphincteric deficiency (ISD) as the needle suspensions offer little to support and coapt the damaged sphincteric unit. It now appears that all patients with stress urinary incontinence probably manifest some degree of ISD and, therefore, simple transvaginal needle suspension will be inadequate for the management of many stress incontinence patients. The role for sling surgery has recently been expanded as these procedures may be performed with less morbidity, while still retaining its high rate of success (3). Therefore, sling surgery is currently more versatile than simple bladder neck suspension as it obviates the need for leak point pressure testing to rule out ISD and if one develops ISD in the future, theoretically, the pubovaginal sling should still correct it. One of the prime objectives now is to perform sling surgery in a minimally invasive fashion, while keeping it successful and readily reproducible.

Since 1992, at the UCLA medical center, we have performed vaginal wall slings on all patients with stress urinary incontinence. Over the past one year, we have employed a new incisionless sling technique which encompasses all the benefits of the anterior vaginal wall sling, yet can be performed with less blood loss and overall less morbidity with excellent initial results.

## MATERIALS AND METHODS

### Patient Selection

A total of 85 patients were evaluated for stress urinary incontinence who eventually underwent the incisionless sling procedure. All patients were evaluated with history, physical exam and multichannel videourodynamics and/or rest and stress voiding cystourethrograms. Basically, all patients with stress incontinence; urethral and bladder neck hypermobility and no evidence of significant anterior vaginal wall prolapse were eligible for this procedure. Patients with anterior vaginal wall prolapse were managed with a similar procedure, yet had a formal four-defect cystocele repair performed.

### Surgical Technique

The first step of the procedure entails placement of a suprapubic catheter. A curved Lowsley retractor is inserted into the urethra and positioned against the anterior bladder wall just above the pubic symphysis and a single stab incision is made down to the retractor. The assistant delivers the catheter into the jaws of the Lowsley and an unlubricated 16Fr catheter is placed. A separate foley catheter is placed in the urethra for the duration of the procedure. Allis clamps are placed just below the urethral meatus to provide upward traction (figure 1). Using a bone drill (Influence Corporation, San Francisco, CA), the operator delivers two separate bone anchors into the inferior aspect of the pubic ramus on either side of the urethra (figure 2). Each anchor is preloaded with a #1 prolene suture. The incision made as the anchor pierces the vaginal epithelium is then widened with a right angle clamp and a subepithelial tunnel is created at the *level of the bladder neck* towards the contralateral side (figure 3). A two by seven cm segment of cadaveric fascia lata is then passed in the tunnel and the prolene suture ends are then passed through the corners of the fascia 5 mm from each end effectively creating a 6 cm sling (figure 4). Cystoscopy is performed to rule out intravesical suture placement and assure satisfactory passage of the suprapubic tube. The fascia is passed through the tunnel and tied in position without regard to tension (figure 5). The incisions are closed with a 2-0 absorbable suture followed by placement of a vaginal pack.

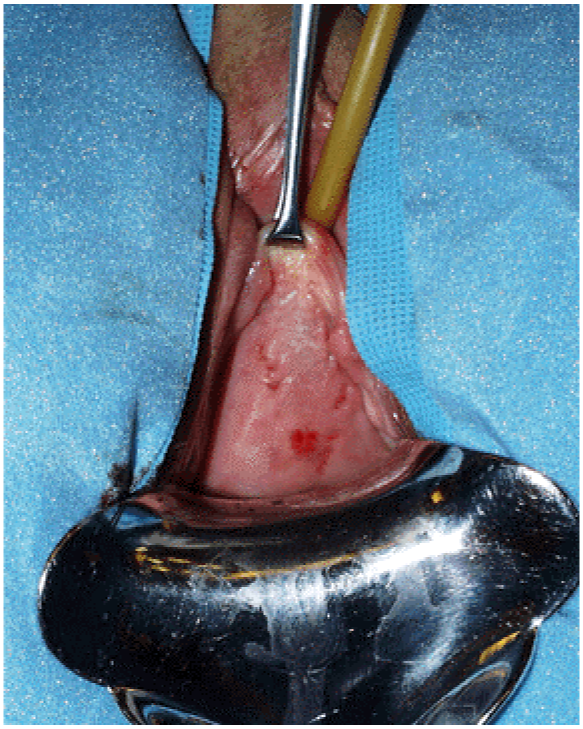

**FIGURE 1.** Allis clamps are placed under the urethral meatus to provide upward traction on the anterior vaginal wall.

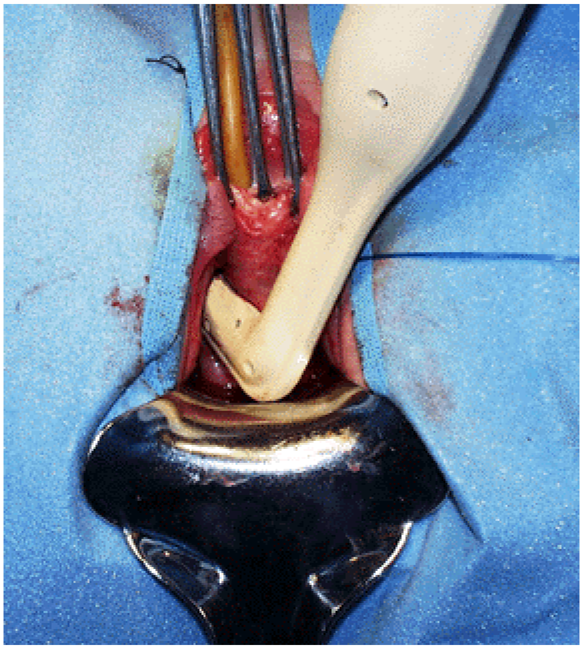

**FIGURE 2.** The bone drill is used to deliver a bone anchor into the inferior aspect of the pubic bone without requiring a vaginal incision. The urethra should be well clear of the drill and the anchor should be secured at the level of the bladder neck.

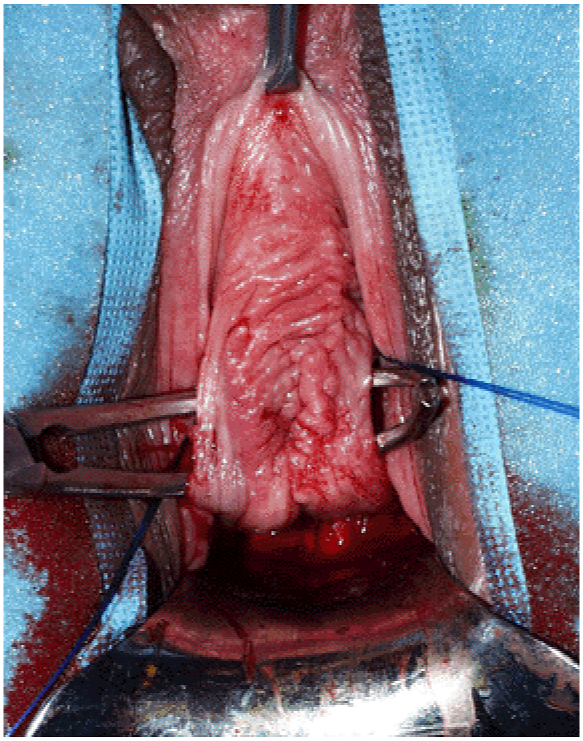

**FIGURE 3**. The incision made as the anchor pierces the vaginal epithelium is widened with a right angle clamp. A subepithelial tunnel is created at the level of the bladder neck towards the contralateral side. Small pockets are created towards the pubic bone such that the excess sling material can reside easily.

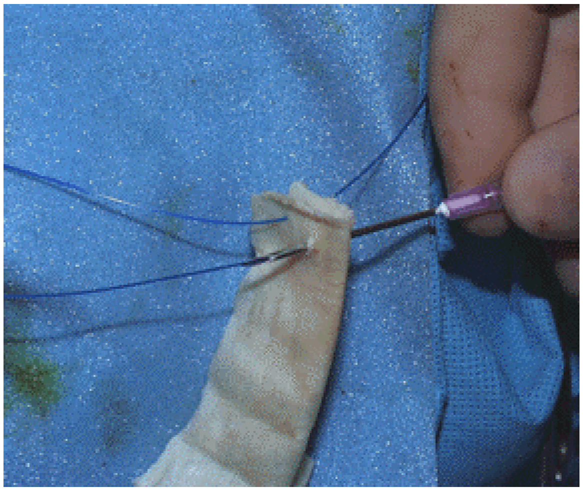

**FIGURE 4**. A 4 x 7 cm segment of cadaveric fascia lata (folded lengthwise to 2 x 7 cm) is then passed into the tunnel and using an 18 gauge needle, the prolene suture ends are passed through the corners 5.0 mm from each end effectively creating a 6.0 cm sling. The sutures are then tied in position.

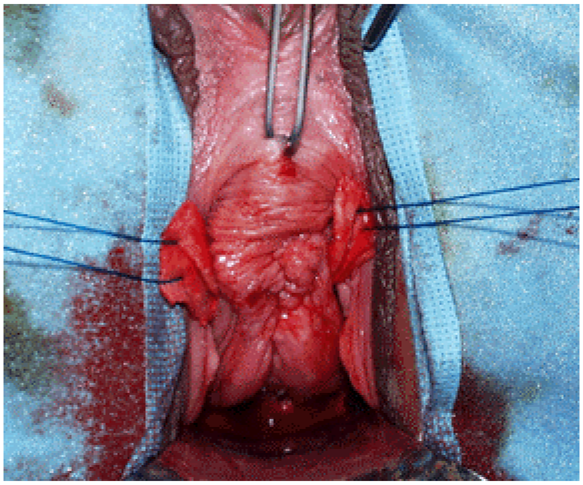

**FIGURE 5**. Sling in place prior to tying sutures. Vaginal incisions are then closed with 2-0 polyglycolic acid sutures and an antibiotic soaked vaginal pack is placed.

## RESULTS

Follow up was obtained by self-administered questionnaire and patient interview. Recurrent SUI was noted in only 4/85 patients (5%). New onset urge incontinence occurred in 2/85 patients (3%). To date, there have been no cases of permanent urinary retention and all cases have been performed on an outpatient basis. Additionally, no complications have occurred and no patient has required a blood transfusion.

## DISCUSSION

The goal of pubovaginal sling surgery is to create a hammock under the bladder neck and urethra such that it forms a backboard of support against which the damaged sphincteric unit can coapt. The pubovaginal sling has enjoyed enormous success over the years as it provides this critical support, while also preventing urethral hypermobility and rotational descent of the bladder neck and urethra. Despite the utility of the sling for ISD alone, the majority of patients have bladder neck and urethral hypermobility along with some component of ISD such that a sling may be the most versatile procedure for most cases of stress urinary incontinence. The main limitations of the sling have been the morbidity from either the procedure itself or the pain associated from harvesting autologous fascial tissues. Accordingly, an effort has been undertaken to create a less invasive sling procedure, while retaining its benefits of suburethral support.

In the past at the UCLA Medical center, an anterior vaginal wall sling (AVWS) was performed for all cases of stress urinary incontinence with or without associated ISD. In a recent review, the overall success rate of the AVWS with long-term follow up at 6 years in 425 patients was 93% (4). New onset urge incontinence was present in 11.1% of patients. The incisionless sling was created in order to improve upon the small, yet significant percentage of patients with de novo urge incontinence. Additionally, within the era of cost containment, an operation that was simpler, faster and tolerated better by the patients may be advantageous.

The incisionless sling encompasses the use of transvaginal bone anchors to create a solid and stable point of fixation for the suspension sutures. The anchors are placed into the inferior aspect of the pubic symphysis followed by passage of the prolene sutures through the cadaveric fascia sling itself. When the sutures are tied in place, the fascia forms the backboard of support that is sought after in the ideal sling.

This minimizes the risk of retention as no "suspension" is performed as in other sling techniques, only suburethral support to prevent inappropriate descent of the bladder neck and urethra. The distance between the sutures (i.e. the sling width) has been calculated to be 6.0 cm by experience. Shortening the sling may create obstruction while making a longer sling may not create enough support and risks recurrent stress incontinence and urethral hypermobility. As such, dissection into the retropubic space is not required, only the space must accommodate the excess 5 mm of fascia on each edge.

The use of cadaveric fascia has greatly simplified the operative procedure. The main advantage it offers is that it is a solid sling material that has stood the test of time (5). As compared with synthetic sling materials, it has a much lower risk of infection and erosion (6,7). Additionally, while fascia may be of similar strength to vaginal wall, it is not subject to vaginal hormonal changes or stretch that may occur as part of pelvic floor relaxation that may limit its use (8). Appell et al. cautioned on the use of in situ vaginal wall for leak point pressures less than 50 cm $H_20$ citing better results with fascia or synthetics for lower leak point pressures (9). Current use of autologous fascia usually adds time to the procedure and increased postoperative discomfort to the patient. To date, no reports of infectious or other disease transmission have been documented in the medical literature with use of cadaveric fascia lata (10). Therefore, cadaveric fascia lata appears to be an ideal material for suburethral sling formation as it is safe, efficacious, and easy to use.

The overall success rates of the incisionless sling compare favorably with the AWVS and other sling techniques (11,12). While the follow up is short, the early results are quite encouraging. This minimally invasive technique is readily reproducible and to date has no significant complications. The avoidance of the vaginal and suprapubic incisions thus far has not compromised efficacy and appears to reduce the incidence of urge incontinence. This versatile technique may be used in all patients with urethral hypermobility and/or ISD with stress urinary incontinence. Long-term follow up will establish the efficacy of this novel approach.
